# An injectable and thermosensitive hydrogel with nano-aided NIR-II phototherapeutic and chemical effects for periodontal antibacteria and bone regeneration

**DOI:** 10.1186/s12951-023-02124-6

**Published:** 2023-10-07

**Authors:** Weixiang Wang, Guorong Zhang, Yanyi Wang, Jianchuan Ran, Lin Chen, Zheng Wei, Huihui Zou, Yu Cai, Wei Han

**Affiliations:** 1 Fourth Clinical Division, Nanjing Stomatological Hospital, Affiliated Hospital of Medical School Nanjing University, 30 Zhongyang Road, Nanjing, 210008 China; 2 Department of Orthodontics, Nanjing Stomatological Hospital, Affiliated Hospital of Medical School, Nanjing University, 30 Zhongyang Road, Nanjing, 210008 China; 3 Department of Oral and Maxillofacial Surgery, Nanjing Stomatological Hospital, Affiliated Hospital of Medical School, Nanjing University, 30 Zhongyang Road, Nanjing, 210008 China; 4 Center for Rehabilitation Medicine, Rehabilitation & Sports Medicine Research Institute of Zhejiang Province, Department of Rehabilitation Medicine, Cancer Center, Affiliated People’s Hospital, Zhejiang Provincial People’s Hospital, Hangzhou Medical College, Hangzhou, 310014 Zhejiang China; 5 Pediatric Dentistry, Nanjing Stomatological Hospital, Affiliated Hospital of Medical School, Nanjing University, 30 Zhongyang Road, Nanjing, 210008 China; 6 Central Laboratory of Stomatology, Nanjing Stomatological Hospital, Affiliated Hospital of Medical School, Nanjing University, 30 Zhongyang Road, Nanjing, 210008 China

## Abstract

Periodontitis is a common public health problem worldwide and an inflammatory disease with irregular defect of alveolar bone caused by periodontal pathogens. Both antibacterial therapy and bone regeneration are of great importance in the treatment of periodontitis. In this study, injectable and thermosensitive hydrogels with 3D networks were used as carriers for controlled release of osteo-inductive agent (BMP-2) and Near Infrared Region-II (NIR-II) phototherapy agents (T8IC nano-particles). T8IC nano-particles were prepared by reprecipitation and acted as photosensitizer under 808 nm laser irradiation. Besides, we promoted photodynamic therapy (PDT) through adding H_2_O_2_ to facilitate the antibacterial effect instead of increasing the temperature of photothermal therapy (PTT). Hydrogel + T8IC + Laser + BMP-2 + H_2_O_2_ incorporated with mild PTT (45 °C), enhanced PDT and sustained release of BMP-2. It was present with excellent bactericidal effect, osteogenic induction and biosafety both in vitro and in vivo. Besides, immunohistochemistry staining and micro-CT analyses had confirmed that PTT and PDT could promote bone regeneration through alleviating inflammation state. Altogether, this novel approach with synergistic antibacterial effect, anti-inflammation and bone regeneration has a great potential for the treatment of periodontitis in the future.

**Supplementary Information:**

The online version contains supplementary material available at 10.1186/s12951-023-02124-6.

## Introduction

Periodontitis is a common public health problem worldwide and carries with substantial morbidity, seriously affecting the patient’s chewing, pronunciation and aesthetics [1–3]. It is an inflammatory disease with irregular defect of alveolar bone caused by periodontal pathogens, which is associated with gingival bleeding, deep periodontal pocket, loosening, displacement or even losing of the teeth [4]. Porphyromonas gingivalis (*P. gingivalis*) is the most dominant bacteria in periodontal disease, especially in chronic periodontitis [5]. At present, the routine treatment procedures for periodontitis includes periodontal nonsurgical therapy (supragingival scaling, subgingival scaling and drug therapy), surgical treatment (bone grafting and guided tissue regeneration), repair treatment and periodontal supporting treatment [6–10]. The existing researches or only focus on the antibacterial effect [11–14], or promote periodontal tissue regeneration [15], neglecting the importance of both antibacteria and osteogenesis at the same time. All those procedures couldn’t completely restore the physiological structure of periodontal tissue. Therefore, it is of great scientific and clinical value to explore a new treatment approach for periodontitis with both antibacterial effect and periodontal tissue regeneration.

Phototherapy is kind of method relying on the sunlight or artificial light (infrared, ultraviolet, visible light laser) to prevent and treat diseases [16–20], which mainly includes photothermal therapy (PTT) and photodynamic therapy (PDT). Hyperthermia > 50 °C is usually required to effectively ablate bacteria through PTT, which inevitably causes thermal damage to surrounding normal tissues [21]. Therefore, the development of a mild PTT strategy that can achieve effective eradication of bacteria is highly anticipated and conducive to the clinical translation of phototherapy. In addition, PDT is an emerging non-invasive treatment modus, which consists of three components: photosensitizer, light and radical oxygen singlet (ROS) [22, 23]. When the photosensitizer is irradiated by light, highly active singlet oxygen is produced by the photochemical reaction, which will improve the thermal sensitivity of biofilms by increasing the permeability of bacterial cell membranes and promote the bactericidal efficiency of near-infrared laser irradiation [24–26]. Light in near-infrared region (NIR) can be further divided into the first NIR window (NIR-I, 650–900 nm) and the second NIR window (NIR-II, 900–1700 nm) according to different emission wavelengths. Although photosensitizer in NIR-I have deeper tissue penetration (less than 1 cm) than the visible light, NIR-I does not appear to be an optimal light source for various deep tissue diseases. In recent years, studies have shown that NIR-II light is more advantageous than NIR-I light with deeper tissue penetration, higher upper limit of radiation and greater tissue tolerance [27, 28]. Therefore, NIR-II nanomaterials with mild PTT, PDT and deeper tissue penetration may have potential advantages in the treatment of periodontitis.

In recent years, Bone tissue engineering has brought new hope for the repair of alveolar bone defects in patients with periodontitis, which has three key factors including seed cells, scaffolds and bioactive factors [29, 30]. Particularly, hydrogel has been widely used as the scaffold and drug carrier, presenting with unique advantages: simple preparation, low cost, low toxicity and sustained drug release [31, 32]. Besides, it is easier to be processed into various shapes, which are suitable for the treatment of tissue defects with irregular shapes [33, 34]. In particular, injectable and thermosensitive hydrogels are able to form minimally invasive wounds by simply injecting pre-gel solution to fill tiny tissue lacunae of various shapes, such as alveolar bone defect due to the periodontitis. The scaffolds with osteo-inductive growth factor has attracted extensive attention in bone tissue engineering [35–39]. Of these, the most commonly used is bone morphogenetic protein-2 (BMP-2), which is approved by the U.S. Food and Drug Administration. BMP-2 can enhance the recruitment and angiogenesis of osteoblast precursor cells and exhibits excellent bone-inducing ability [40, 41]. Injectable hydrogels with 3D networks have become the primary choice for BMP-2 carriers in tissue engineering due to their high water-absorption, syringe ability, biocompatibility and biodegradability [42–44]. The combination of hydrogel and BMP-2 has achieved good bone tissue regeneration and repair in those bone defect diseases [45–47].

**Scheme 1:**
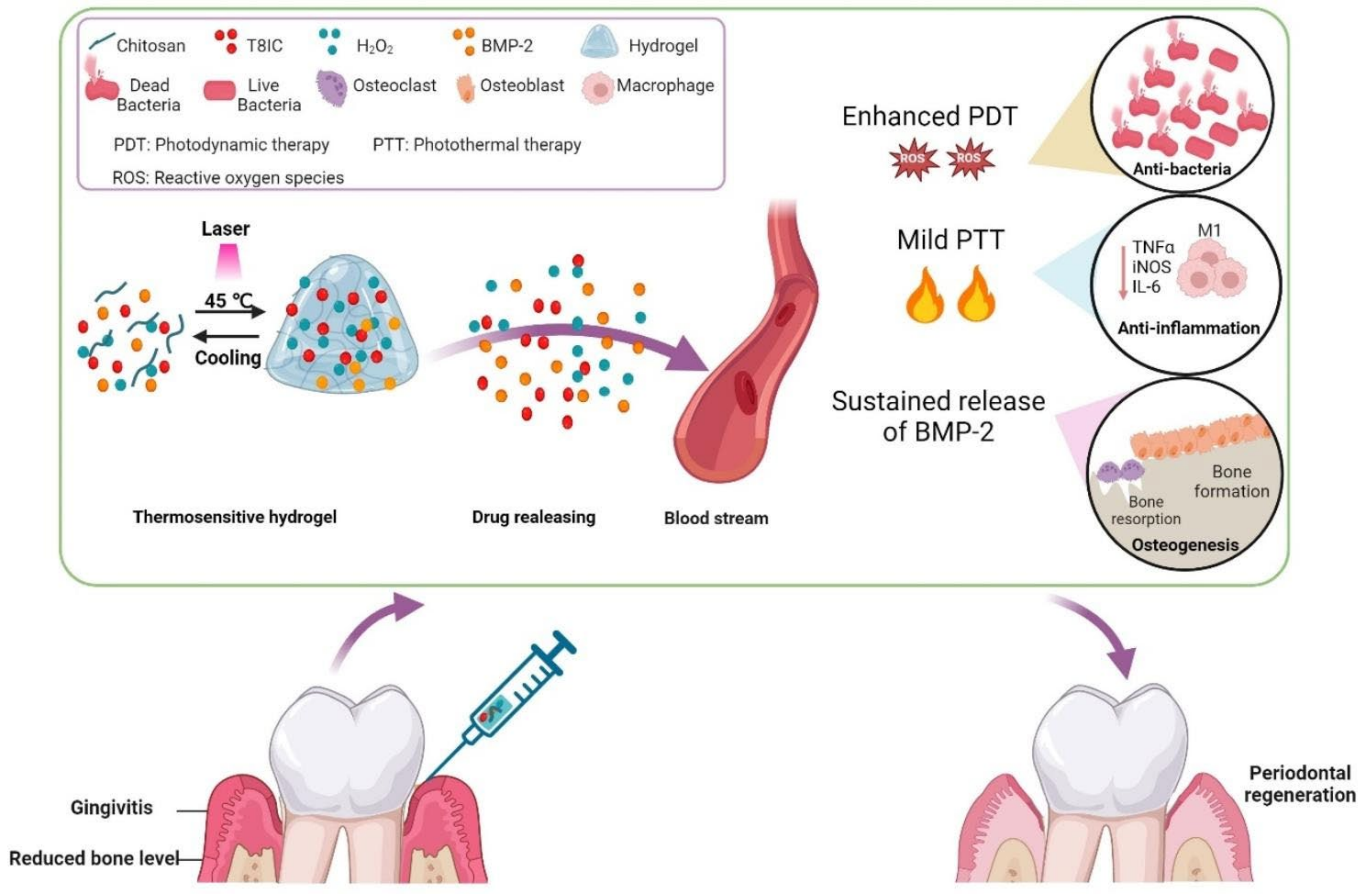
Schematic illustration of thermosensitive and injectable hydrogel with T8IC, H_2_O_2_ and BMP-2. Hydrogel was used as drug carrier for controlled drug release. T8IC combined with H_2_O_2_ exhibited excellent anti-bacteria and anti-inflammation effects through enhanced PDT and mild PTT. Sustained release of BMP-2 promoted periodontal bone regeneration

 [15]Herein, an injectable and thermosensitive hydrogel with 3D networks was used as a carrier for controlled release of osteo-inductive agent (BMP-2) and NIR-II phototherapy agents (T8IC and H_2_O_2_). Hydrogel + T8IC + Laser + BMP-2 + H_2_O_2_ incorporated with mild PTT (45 °C), enhanced PDT and sustained release of BMP-2, which exhibited excellent bactericidal effect, osteogenic induction and biosafety in vitro and in vivo. Besides, it was confirmed that PTT and PDT could promote bone regeneration through alleviating inflammation state. Altogether, this novel approach with synergistic antibacterial effect, anti-inflammation and bone regeneration has a great potential for the treatment of periodontitis in the future (Scheme 1).

## Materials and methods

### Synthesis and characterizations of hydrogel + T8IC + laser + BMP-2

1.5 mg T8IC (C_90_H_76_N_4_O_2_S_8_) was dissolved in 1 ml tetrahydrofuran (THF) and 1.5 mg DSPE-mPEG2000 was dissolved in 5 ml phosphate buffer saline (PBS). Then, the T8IC solution was dripped into DSPE-mPEG2000 solution under magnetic stirring (800 rpm) at room temperature. After stirring for 5 min, the mixed solution was removed to rotary evaporation. When the solution was dried, 5 mL PBS was added and T8IC nano-particles (T8IC NPs) were obtained by reprecipitation under ultrasonic shaking for 10 min. The size and morphology of T8IC NPs were detected by dynamic light scattering (DLS) and transmission electron microscope (TEM). UV-visible light absorption, NIR-II fluorescence emission were tested by the UV-Vis spectrophotometer.

After 3 min UV irradiation, 0.8 g chitosan was dissolved in 40 mL of sterile 0.1 mol/L HCl solution. 5 mg gelatin was dissolved in 1mL of deionized water at room temperature and filtered through a 0.22 μm filter. Subsequently, 2 g β-glutamine was dissolved in 8 mL deionized water and filtered through a 0.22 μm filter. Then, 40 mL of chitosan solution was mixed with 1 mL of gelatin solution and 8 mL of β-glutamine solution under magnetic stirring (800 rpm). 0.1 mol/L NaOH solution was added into the mixture by drop until the pH was 7.0. Finally, T8IC NPs (40 µg/ml) obtained by reprecipitation [48] and BMP-2 (100 ng/ mL) were added into hydrogel under magnetic stirring (800 rpm). The morphology of Hydrogel + T8IC + Laser + BMP-2 was observed by scanning electron microscopy after lyophilized (-50 ℃, 24 h). The viscosity was detected by a viscometer. Hydrogel + T8IC + Laser + BMP-2 was dissolved in the PBS to detect the sustained drug release. The photothermal effect under different laser power (2, 1.5 and 1 W/cm^2^) and different concentrates of T8IC NPs (60, 40, 20, 0 µg/ml, 1 W/cm^2^) and photostability (60 µg/ml, 2 W/cm^2^) were characterized and recorded by an infrared camera.

### Cytotoxicity of hydrogel + T8IC + laser + BMP-2

MC3T3-E1 cell line, a mouse skull osteoblast precursor cell line, was purchased from Shanghai Zhongqiao Xinzhou Biotechnology Co. LTD. The effects of different concentrations of T8IC NPs without laser irradiation and different temperatures (37 ℃, 45 ℃, 50 ℃ and 55 ℃, generated by Hydrogel + T8IC + Laser + BMP-2) on the proliferation of MC3T3-E1 cells were detected through CCK-8 kit. The actin of MC3T3-E1 cells was labeled with Rhodamine-Phalloidin and the nucleus was labeled with DAPI to observe the effect of different treatments on the cytoskeleton of MC3T3-E1 cells.

### Reactive oxygen species (ROS) generation

The generation of ROS in the two groups: Hydrogel + T8IC + Laser + BMP-2, Hydrogel + T8IC + Laser + BMP-2 + H_2_O_2_ was monitored by the oxidation of DPBF at 418 nm in dichloromethane.

### Bacterial eradication of hydrogel + T8IC + laser + BMP-2 + H_2_O_2_

*P. gingivalis* (ATCC 33,277) was cultured in brain heart infusion (BHI) medium with yeast extract (50 mg/L), hemin (1 mg/L) and vitamin K3 (1 mg/L) in a humidified incubator at 37 °C anaerobically. *P. gingivalis* was inoculated in the lower chamber of a 96-well plate (10^6^ CFU/ml, 100 ul/well) and the hydrogel was placed in the upper chamber of a transwell 96-well plate and irradiated with an 808 nm laser (1.5 W/cm^2^) for 4 min: Control, Hydrogel, Hydrogel + H_2_O_2_ (0.15%), Hydrogel + BMP-2 (100 ng/ mL), Hydrogel + T8IC + Laser (45 ℃, 40 ug/ml T8IC, 1.5 W/cm^2^), Hydrogel + T8IC + Laser + BMP-2 + H_2_O_2_ (45 ℃, 40 ug/ml T8IC, 100 ng/ mL BMP-2, 1.5 W/cm^2^, 0.15% H_2_O_2_). The proliferation of *P. gingivalis* was detected by the optical density (OD) at 600 nm and 1 was corresponding to 10^9^ CFU/ml. At the same time, the bacterial of each group was diluted 2 × 10^4^ times and one drop of the bacteria solution was evenly spread on the blood plate for colony culture. After 10 days, the colony forming units (CFU) of each group were counted. The biofilm live/dead staining in each group was also observed via confocal laser scanning microscopy. The live bacteria were stained with SYT09 dye (5 µM, green fluorescence) and the dead bacteria was labeled with PI (8 µM, red fluorescence).

### Quantitative real-time PCR

MC3T3-E1 cells were seeded in a 6-well plate (2 × 10^5^/well) and cells in each group (Control, Hydrogel, Hydrogel + H_2_O_2_ (0.15%), Hydrogel + BMP-2 (100 ng/mL), Hydrogel + T8IC + Laser (40 ug/ml T8IC, 1.5 W/cm^2^, 45 ℃), Hydrogel + T8IC + Laser + BMP-2 + H_2_O_2_ (40 ug/ml T8IC, 100 ng/ mL BMP-2, 1.5 W/cm^2^, 0.15% H_2_O_2_, 45 ℃,) were separately cultured in osteogenic induction medium for 4 days. MC3T3-E1 cells were inoculated in the lower chamber of a 6-well plate and the hydrogel was placed in the upper chamber of a transwell 6-well plate.

After 4 days of culture, the cells were rinsed 3 times with PBS and total RNA was extracted according to the instructions of QIAzol reagent. RNA was quantified by Nanodrop Spectrophotometer and reverse-transcribed by Superscript II (Takara, Japan). The mRNA expression of osteogenesis related genes (Runx 2, ALP, OCN, OPN and Col-I) in each group was detected by quantitative real-time PCR using SYBR Green Master MIX (ABI, USA). GAPDH was used as internal reference for mRNA level and x = 2^−ΔΔCT^ was used for quantitative analysis of gene expression.

### ALP activity, ALP staining and alizarin red staining

The osteogenic differentiation ability of MC3T3-E1 cells was detected by alkaline phosphatase (ALP) activity kit (4 days and 7 days), ALP staining kit (4 days) and alizarin red staining kit (21 days), respectively.

### Metabolism and degradation of Hydrogel + T8IC + Laser + BMP-2 + H_**2**_**O**_**2**_ in vivo

All animals were kept in a pathogen-free environment and fed ad lib. The procedures for care and use of animals were approved by the Ethics Committee of Nanjing University and all applicable institutional and governmental regulations concerning the ethical use of animals were followed. After subcutaneous injection in the back and 808 nm laser irradiation for 4 min were applied, the distribution and degradation of Hydrogel + T8IC + Laser + BMP-2 + H_2_O_2_ and T8IC NPs in the body of BALB/C nude mice (0, 1, 2, 5, 10 and 21 days) was observed by an in vivo fluorescence detector with near-infrared second-window fluorescence scanner. After 3 weeks, the mice in each group were euthanized.

### Animal periodontitis model

The maxillary second molars of female C57BL/6J mice were ligated with 5 − 0 silk thread (immersed in *P. gingivalis* bacterial suspension for 12 h) to establish a periodontitis model [49]. Two weeks later, micro-CT analysis was performed in each group to observe whether the periodontitis model was successfully established. Then, the mice were randomly divided into six groups with five mice in each group: Control, Periodontitis, Hydrogel, Hydrogel + T8IC + Laser (40 ug/ml T8IC, 1.5 W/cm^2^), Hydrogel + BMP-2 (100 ng/ml BMP-2), Hydrogel + T8IC + Laser + BMP-2 + H_2_O_2_ (40 ug/ml T8IC, 1.5 W/cm^2^, 100 ng/ mL BMP-2, 0.15% H_2_O_2_). Drugs in each group were slowly injected into the periodontal pocket of the maxillary second molars.

### Blood routine test and blood biochemical examination

After 6 weeks, the C57BL/6J mice in each group were euthanized and peripheral blood was obtained by removing the eyeballs. Blood routine (white blood cell count WBC, red blood cell count RBC, hemoglobin determination Hb and hematocrit HCT), liver function (total bilirubin TBIL, albumin Alb, globulin GLB, alanine aminotransferase ALT and aspartate aminotransferase AST, alkaline phosphatase ALP and glutamyl transpeptidase GGT) and renal function (urea BUN, creatinine CRE and uric acid UA) were detected.

### Micro-computerized tomography (Micro-CT) scanning

Micro-CT was used to detect alveolar bone defect repair of C57BL/6J mice. After euthanized, the maxilla with molars were fixed in 4% paraformaldehyde overnight and then were examined by a viva CT micro-computed tomography scanner (Scanco Medical, Bassersdorf, Switzerland) at 10.5 μm resolution. The 3D images of the maxilla were reconstructed. The distance between cemento-enamel junction and the alveolar bone crest (CEJ-ABC) was measured to evaluate the periodontal defect. Volumetric and morphometric analyses including bone mineral density (BMD, mg/cm^3^) and bone volume fraction (bone volume/tissue volume, BV/TV, %) were also performed.

### Immunohistochemical and H&E staining

Immunohistochemical staining was used to observe the expression of inflammatory factors (proinflammatory factors IL-6, TNF-α and iNOS) in the periodontal tissue of C57BL/6J mice. After treatment, the main organs (heart, liver, spleen, lung and kidney) were dissected from the mice. HE staining was performed to observe the inflammatory status of the tissues and the changes of cell morphology.

### Statistical analysis

All the quantitative results were reported as mean ± standard deviation from at least three independent studies. The statistical analysis was performed by using the statistical software (SPSS 22.0, America). Multiple comparisons of data were performed by two-tailed Student’s t tests or one-way analysis of variance. Probability value of P < 0.05 was considered statistically significant.

## Results and discussion

### Synthesis and characterizations of hydrogel + T8IC + laser + BMP-2

T8IC is a novel organic semiconducting material, mainly used in high performance organic photovoltaic device. T8IC nano-particles (T8IC NPs) were synthesized through reprecipitation. The Zeta potential of T8IC NPs was 21.9 ± 1.12 mV. It was homogeneously distributed and kept stable in PBS and DMEM for 14 days (Figure S1A). The absorption spectrum of T8IC NPs was 600 ~ 900 nm (Figure S1B). It belonged to the second near-infrared region spectrum (NIR-II, 900 ~ 1700 nm), of which the emission fluorescence spectrum was 900 ~ 1100 nm (Figure S1C). The TEM image exhibited spherical morphology of T8IC NPs (Figure S1D). The average diameter of T8IC NPs was about 160 nm (Figure S1E-S1F). Besides, T8IC NPs could produce ROS after 808 nm laser irradiation, suggesting a good potential for PDT [48]. Both PTT and PDT of T8IC NPs exhibited good tumor elimination when combined with sorafenib in our previous study [48].

**Fig. 1:**
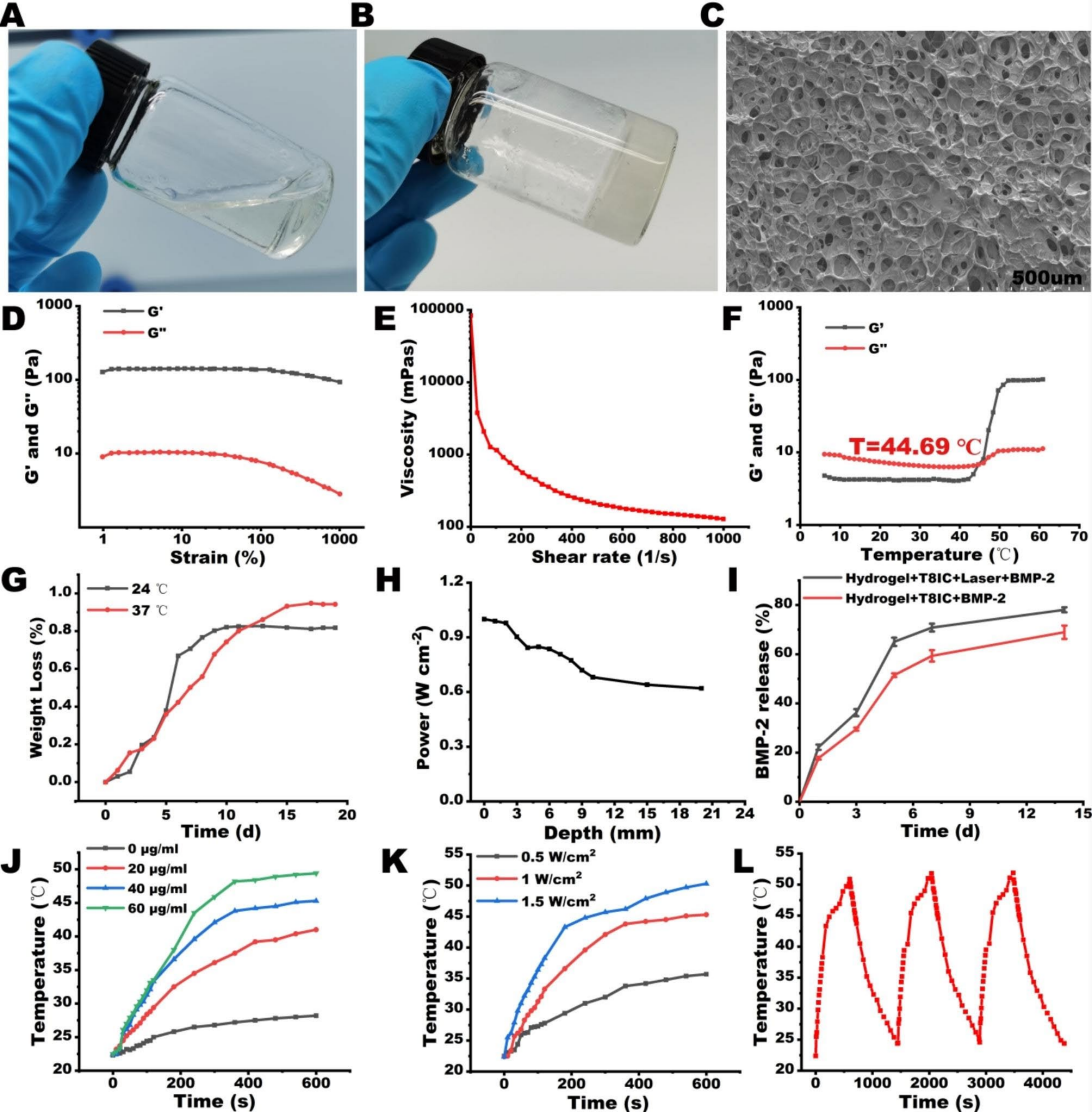
Synthesis and characterizations of Hydrogel + T8IC + Laser + BMP-2. **A-B**: Representative photographs of sol-gel transition. **C**: Scanning electron microscopy of Hydrogel + T8IC + Laser + BMP-2. **D**: Variations of storage and loss moduli (G’ and G’’, respectively) *versus* strain (%). **E**: Viscosity *versus s*hear rate (1/s). **F**: The temperature dependent oscillatory rheology experiment. **G**: The weight loss (%) of Hydrogel + T8IC + Laser + BMP-2. **H**: Laser (808 nm) depth penetration of Hydrogel + T8IC + Laser + BMP-2. **I**: Cumulative release of BMP-2. **J**: Photothermal effects of Hydrogel + T8IC + Laser + BMP-2 with different concentration of T8IC NPs. **K**: Photothermal effects at different laser power. **L**: Photothermal stability of Hydrogel + T8IC + Laser + BMP-2.

The translucent chitosan/β-glutamine/gelatin pre-gel was injectable and flowed like a viscous liquid. Interestingly, the sol-gel transition occurred when the temperature was around 45 ℃ within 2 minutes (Fig. 1A-1B). Scanning electron microscope (SEM) images revealed porous structure within the freeze-dried hydrogel (Fig. 1C), which facilitated the storage and release of drugs and cell migration [15]. The rheological properties of Hydrogel + T8IC + Laser + BMP-2 was investigated through rheological tests. The strain dependent oscillatory rheology experiments (Fig. 1D) revealed that the storage modulus (G’) and the loss modulus (G’’) remained constant when strain was below 100% while the frequency was 1 Hz. Furthermore, the G’ was over 100 Pa and much higher than the G’’, indicating stable hydrogel networks formed. In addition, the viscosity of hydrogel decreased sharply with increasing shear rate (Fig. 1E), implying a shear thinning property, which is beneficial for further injection [50]. The temperature dependent oscillatory rheology experiment (Fig. 1F) was also conducted and when the temperature was below 44.69 ℃, the value of G’ and G’’ was stable and G’’ was higher than that of G’. While modulus of G’ and G’’ increased with the temperature sharply and the G’ and G’’ crossed at a temperature of 44.69 ℃. Then, the value of G’ was higher than that of G’’, implying the sol-gel transition. Thus, such thermosensitive hydrogel is easier to be injected into bone defects with irregular shapes due to the periodontitis. The weight loss (%) of Hydrogel + T8IC + Laser + BMP-2 increased with time at room temperature (24 ℃) and 37 ℃ (Fig. 1G). 82% weight loss at room temperature (24 ℃) after 10 days and 93% weight loss at 37 ℃ after 14 days. In order to evaluate the effective laser (808 nm) depth penetration of the hydrogel, the residual laser intensity was detected along with the hydrogel depth (Fig. 1H). The laser intensity decreased with the hydrogel depth. 62% residual laser intensity was detected when the hydrogel depth was 20 mm, indicating that hydrogel combined with T8IC NPs was capable for the treatment of periodontitis with deep periodontal pocket.

The hydrogel has been widely used as the scaffold and drug carrier for controlled drug release in craniofacial tissue engineering, contributing to delivering antibiotics, growth factors, chemotherapy drugs, vaccines and anti-inflammatory agents to aim tissues and cells [51–53]. Dual-functional delivery systems present with synergistic effects including inhibiting bacterial growth, facilitating wound healing, reinforcing bone regeneration and reducing inflammatory responses. In the present study, osteo-inductive agent (BMP-2) and NIR-II phototherapy agents (T8IC NPs) were applied within the hydrogel. The BMP-2 releasing from the hydrogel (Fig. 1I) had been monitored. Cumulative BMP-2 release of Hydrogel + T8IC + Laser + BMP-2 was higher than that of Hydrogel + T8IC + BMP-2.

A good photothermal conversion ability of Hydrogel + T8IC + Laser + BMP-2 was detected under different concentrations of T8IC NPs and different laser power (808 nm) (Fig. 1J K). The photothermal effect was concentration and power dependent. It was also presented with a good photothermal stability (Fig. 1L).

### Photothermal effects on the cell proliferation in vitro

**Fig. 2:**
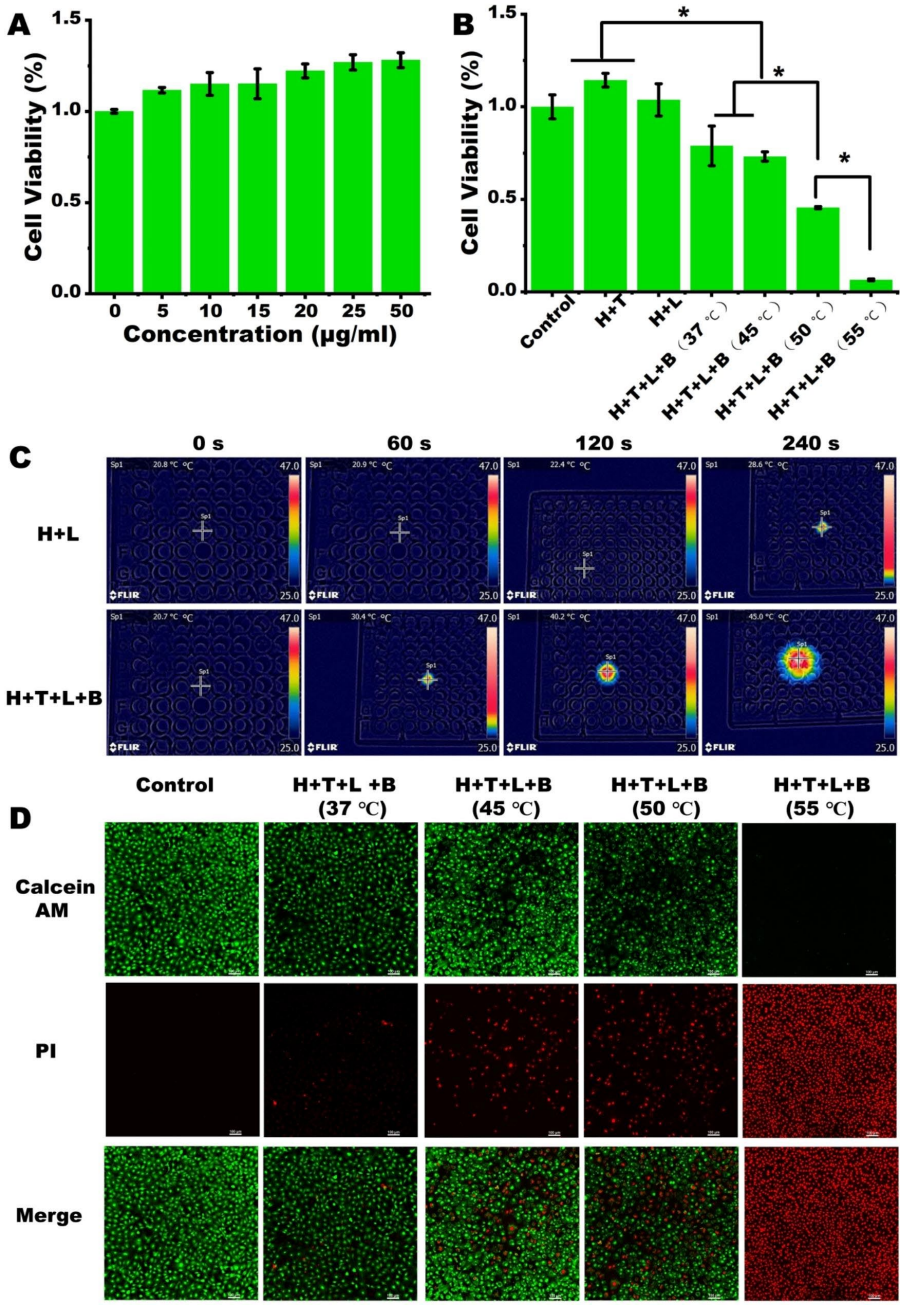
Photothermal effects on the proliferation of MC3T3-E1 cells. **A**: Different concentrations of T8IC NPs on the proliferation of MC3T3-E1 cells. **B**: Photothermal effect on the proliferation of MC3T3-E1 cells, H: hydrogel, T: T8IC, L: Laser, B: BMP-2. **C**: Photothermal effects in 96-well plates. **D**: The Live-dead cell staining of MC3T3-E1 cells (green: live cells, red: dead cells), scale bar: 100 μm

In this study, the cytotoxicity of T8IC NPs was assessed through CCK-8 assay. The cell viability of MC3T3-E1 cells wasn’t influenced by T8IC NPs when the concentration was under 50 µg/ml without irradiation (Fig. 2A), implying a good biosafety. As for the photothermal effects, Hydrogel + T8IC + Laser + BMP-2 exhibited remarkable cell toxicity with 808 nm laser irradiation (1.5 W/cm^2^) when the temperature was over 45 ℃. The cell viability decreased to 73%, 45%, 6% after temperature reached to 45 ℃, 50 ℃ and 55 ℃, respectively (Fig. 2B). Photothermal effect of Hydrogel + T8IC + Laser + BMP-2 was also monitored in 96-well plates (Fig. 2C). The temperature of Hydrogel + T8IC + Laser + BMP-2 raised to 45 ℃ within 4 min, while it was only up to 28.6 ℃ in the Hydrogel + Laser group. The live/dead cell staining also proved that the higher temperature was and more red-fluorescence detected, implying less cells alive (Fig. 2D). Thus, a relative mild temperature should be chosen to protect cells from the hyperthermia and also meet the need of bactericidal effect in the subsequent study.

### Antibacterial effect in vitro

With high prevalence of antibiotic-resistant bacteria, many novel nano-material based antibacterial strategies has been created at the right time [53, 54]. However, the side effect of high temperature created by PTT on the surrounding tissue may limit its clinical application. Thus, it is meaningful to seek a mild temperature which can kill the bacterial and also reduce the damage to the surrounding tissue. Many studies have elucidated that mild temperature PTT can achieve fantastic therapeutic performance to enable practical biomedical applications. Although there are different thresholds of mild temperature PTT, it is widely recognized that most of the mild hyperthermia treatments are conducted below 48 ℃ [55].

**Fig. 3:**
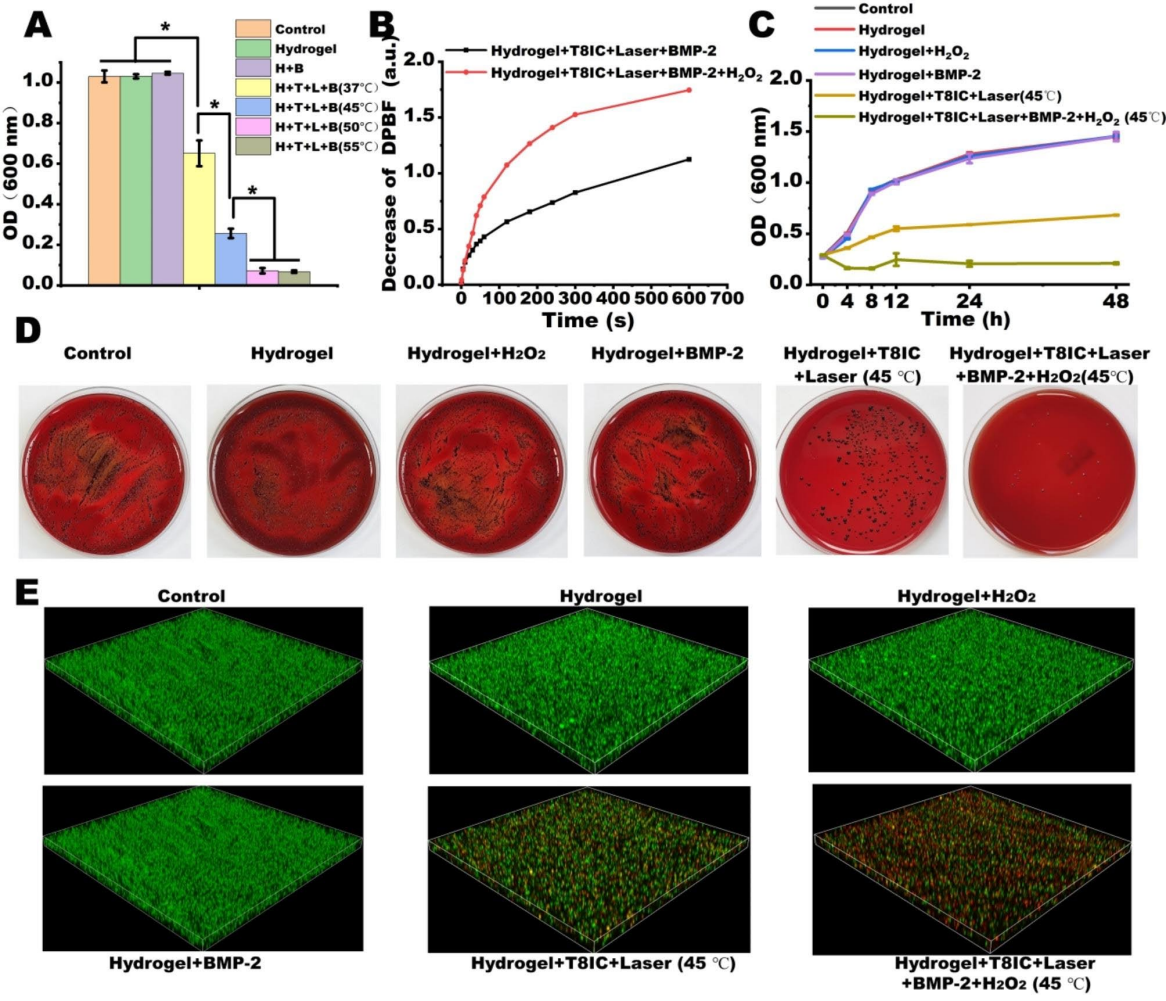
The antibacterial effect in vitro. **A**: OD value of *P. gingivalis*. at 600 nm. H: hydrogel, T: T8IC, L: Laser, B: BMP-2. **B**: ROS generation after adding H_2_O_2_, s: second. **C**: OD value of *P. gingivalis*. at 600 nm under different conditions, h: hour. **D**: Photographs of *P. gingivalis* colonies after different treatments. **E**: 3D images of live/dead bacterial staining (green: live cells, red: dead cells) after different treatments

Firstly, we assessed the temperature on the bacterial inhibition. The hydrogel and Hydrogel + BMP-2 had no effect on the *P. gingivalis* proliferation (Fig. 3A). The bacterial number decreased at a control group of 37 ℃ after laser irradiation, indicating that PDT plays an important role in the bacterial ablation. Besides, the eradication rates of pathogens increased with the temperature. The antibacterial activity of Hydrogel + T8IC + Laser + BMP-2 (45 ℃) was enhanced compared with Hydrogel + T8IC + Laser + BMP-2 (37 ℃), but still not enough to completely eliminate biofilms. Thus, we planned to promote the PDT to facilitate the antibacterial effect instead of increasing the temperature, which was harmful to the cell proliferation. Occasionally, H_2_O_2_ was found to be a good photosensitizer, which was commonly used as periodontal pocket rinse solution after periodontal initial therapy. Although H_2_O_2_ can effectively kill bacteria by attacking and destroying DNAs and proteins, a high concentration of H_2_O_2_ exhibited toxic effect to the normal tissue. After catalyzed into reactive oxygen species (ROS), H_2_O_2_ manifested better antibacterial efficiency [25, 56]. Therefore, a lower concentration of H_2_O_2_ and enhanced antibacterial efficiency was designed. DBPF probe was used to assess changes of ROS content in Hydrogel + T8IC + Laser + BMP-2 and Hydrogel + T8IC + Laser + BMP-2 + H_2_O_2_ under 808 nm laser irradiation. As shown in Fig. 3B, the ROS yield was calculated by monitoring the oxidation of DPBF at 418 nm in dichloromethane [57]. The more DBPF loss after adding H_2_O_2_, implying more ROS generated and a better PDT effect. Other studies had shown that the combination of PDT and PTT could destroy the bacterial membranes and enhance bacterial eradication [14, 58, 59]. As a result, the antibacterial effect had been promoted in the Hydrogel + T8IC + Laser + BMP-2 + H_2_O_2_ (45 ℃) group compared to the Hydrogel + T8IC + Laser (45 ℃) group (Fig. 3C), suggesting that synergistic effect of an enhanced PDT and a mild temperature PTT (45 ℃) could acquire a better bacterial eradication. There was no significant difference among control, Hydrogel, Hydrogel + H_2_O_2_ and Hydrogel + BMP-2 after 10 days culture by the colony forming unit assay (Fig. 3D). Both Hydrogel + T8IC + Laser (45 ℃) and Hydrogel + T8IC + Laser + BMP-2 + H_2_O_2_ (45 ℃) had significantly less *P. gingivalis* colonies, while the pathogens in the Hydrogel + T8IC + Laser + BMP-2 + H_2_O_2_ (45 ℃) were nearly entirely eliminated. In addition, *P. gingivalis* suspended within the BHI medium, so 3D images of the live/dead (green/red) bacterial fluorescence staining were built up to detect the biofilm viability of *P. gingivalis* (Fig. 3E). Absolutely only green fluorescence was observed in the group of control, Hydrogel, Hydrogel + H_2_O_2_ and Hydrogel + BMP-2, indicating biofilms were intact. A small amount of red fluorescence was detected in Hydrogel + T8IC + Laser (45 ℃) and the biofilms were almost stained red in Hydrogel + T8IC + Laser + BMP-2 + H_2_O_2_ (45 ℃), consistent with the colony forming unit assay. In general, the phototherapy generated from Hydrogel + T8IC + Laser + BMP-2 + H_2_O_2_ including enhanced PDT and mild PTT, exhibited a superior bactericidal effect. Therefore, we finally selected 45 ℃ as a mild temperature in the following study.

### Osteogenic effect in vitro

**Fig. 4:**
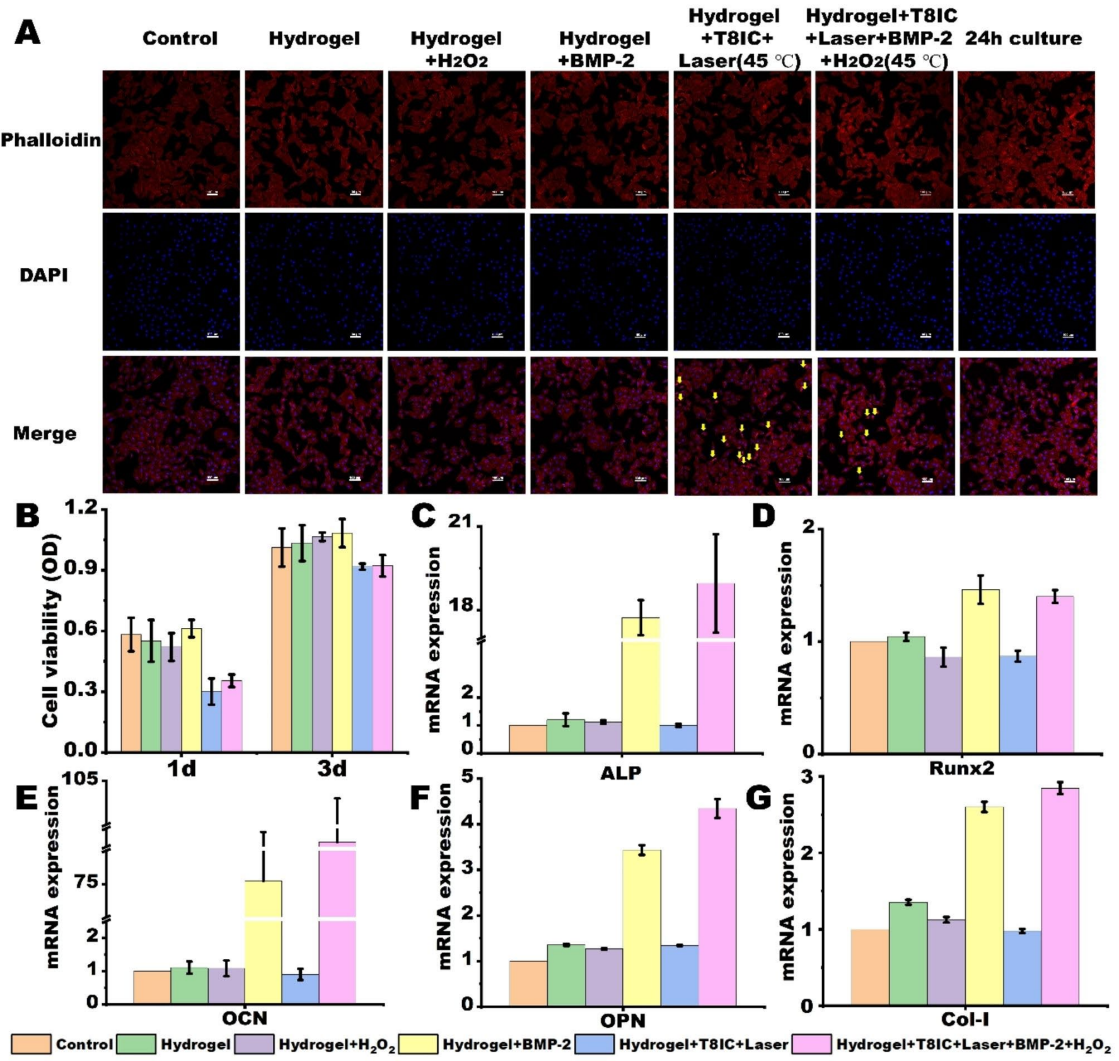
Cytotoxicity and osteogenic effect in vitro. **A**: The changes of cytoskeleton after different treatments, yellow arrow: cell shrinkage, scale bar: 100 μm. **B**: Cell viability 1 day and 3 days after different treatments. **C-G**: mRNA expression of osteogenic marker (ALP, Runx2, OCN, OPN, Col-I).

In order to detect the effect of enhanced PDT and mild PTT to the cell proliferation, the cell spreading of MC3T3-E1 cells after different treatments was monitored. As shown in Fig. 4A, Hydrogel, Hydrogel + H_2_O_2_ and Hydrogel + BMP-2 had less effect on the actin filaments labelled by phalloidine (red), while cell shrinkage and detachment in the Hydrogel + T8IC + Laser (45 ℃) and Hydrogel + T8IC + Laser + BMP-2 + H_2_O_2_ (45℃) were observed due to the combined effects of PTT and PDT. Delightfully, the cell regained the bioactivity, cell spreading and attachment after another 24-hour culture. Besides, although the proliferation of MC3T3-E1 cells was inhibited in Hydrogel + T8IC + Laser and Hydrogel + T8IC + Laser + BMP-2 + H_2_O_2_ 1 day after treatment, the cell viability had resuscitated 3 days after treatment (Fig. 4B).

Although BMP-2 enhances the recruitment and angiogenesis of osteoblast precursor cells and has attracted much attention for its excellent bone-inducing ability [40, 41], its commercial use is limited by two major aspects: firstly, BMP-2 has a short half-life (only 7 min) in the physiological environment and a high dose of BMP-2 is required to be loaded into those scaffolds to solve its instability and rapid inactivation, which may result in significant costs and an increased risk of side effects, including inflammation, nerve damage, ectopic ossification and tumorigenesis [60–62]. Secondly, absorbable collagen sponge and calcium phosphate [63, 64] are currently the only two BMP-2 carriers approved for clinical use. However, because of the low affinity of these vectors for BMP-2 and their low retention rate, the use of high doses can also exacerbate local and systemic adverse effects. To overcome these problems and optimize the bone healing process, various approaches have focused on improving the loading rate of BMP-2 while maintaining its biological activity. Among them, injectable hydrogels with 3D networks have become the primary choice for BMP-2 carriers in tissue engineering due to their high water-absorption, injectable ability, encapsulation ability, biocompatibility and biodegradability [42–44].

Osteoblast differentiation was assessed by mRNA expression (**Figure S2**), quantitative analysis of ALP activity, ALP staining and alizarin red staining. After 4 days culture, gene expression of osteogenic mRNAs (early osteogenic marker: ALP, specific osteogenic differentiation markers in the earlier stage: Runx2, late osteogenic marker: OCN, non-collagenous proteins: OPN, extracellular matrix protein: Col-I) was much higher in the Hydrogel + BMP-2 and Hydrogel + T8IC + Laser + BMP-2 + H_2_O_2_ (Fig. 4C-G, P < 0.05). While the mRNA expression (ALP, OCN and OPN) of Hydrogel + T8IC + Laser + BMP-2 + H_2_O_2_ was significantly higher than that of Hydrogel + BMP-2 (P < 0.05), indicating that PTT could induce the release of BMP-2 and promote osteogenic gene expression, as shown in Fig. 1I.

**Fig. 5:**
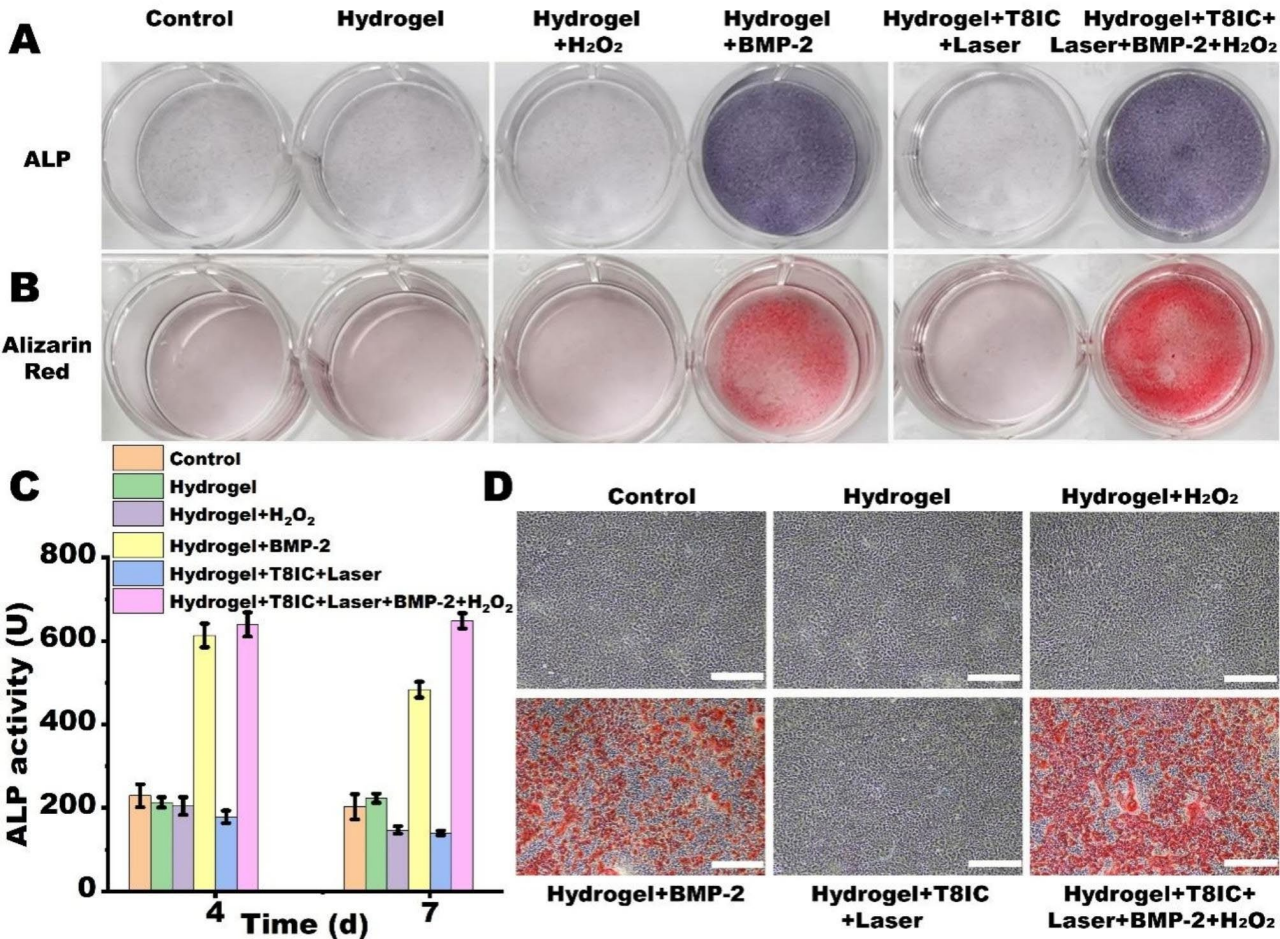
The osteogenic effect in vitro. **A**: ALP staining. **B**: Alizarin red staining. **C**: ALP activity. **D**: Microscopic photographs of alizarin red staining, red: deposition of mineralized nodules, scale bar :100 μm

The ALP staining (Fig. 5A**)** and alizarin red staining (Fig. 5B**)** were consistent with the results of PCR. Hydrogel + BMP-2 and Hydrogel + T8IC + Laser + BMP-2 + H_2_O_2_ exhibited intensified staining of ALP and mineralized nodules. In addition, the ALP activity of Hydrogel + BMP-2 and Hydrogel + T8IC + Laser + BMP-2 + H_2_O_2_ was much higher than other groups after both 4 days and 7 days culture (Fig. 5C, P < 0.05). The ALP activity of Hydrogel + T8IC + Laser + BMP-2 + H_2_O_2_ after 7 days culture was higher than that of Hydrogel + BMP-2. Furthermore, the microscopic photographs of alizarin red staining showed there was highest density of mineralized nodules deposition in the Hydrogel + T8IC + Laser + BMP-2 + H_2_O_2_ (Fig. 5D**).** All those results proved that the mild PTT and enhanced PDT generated by Hydrogel + T8IC + Laser + BMP-2 + H_2_O_2_ may not affect the release and bioactivity of BMP-2. On the contrary, as shown in Fig. 1I, the increase of local temperature may destroy hydrogen bond interactions and accelerate hydrogel degradation to increase the release of BMP-2 and bone-inducing activity [65].

### Toxicity and metabolism of Hydrogel + T8IC + Laser + BMP-2 + H_**2**_**O**_**2**_ in vivo

**Fig. 6:**
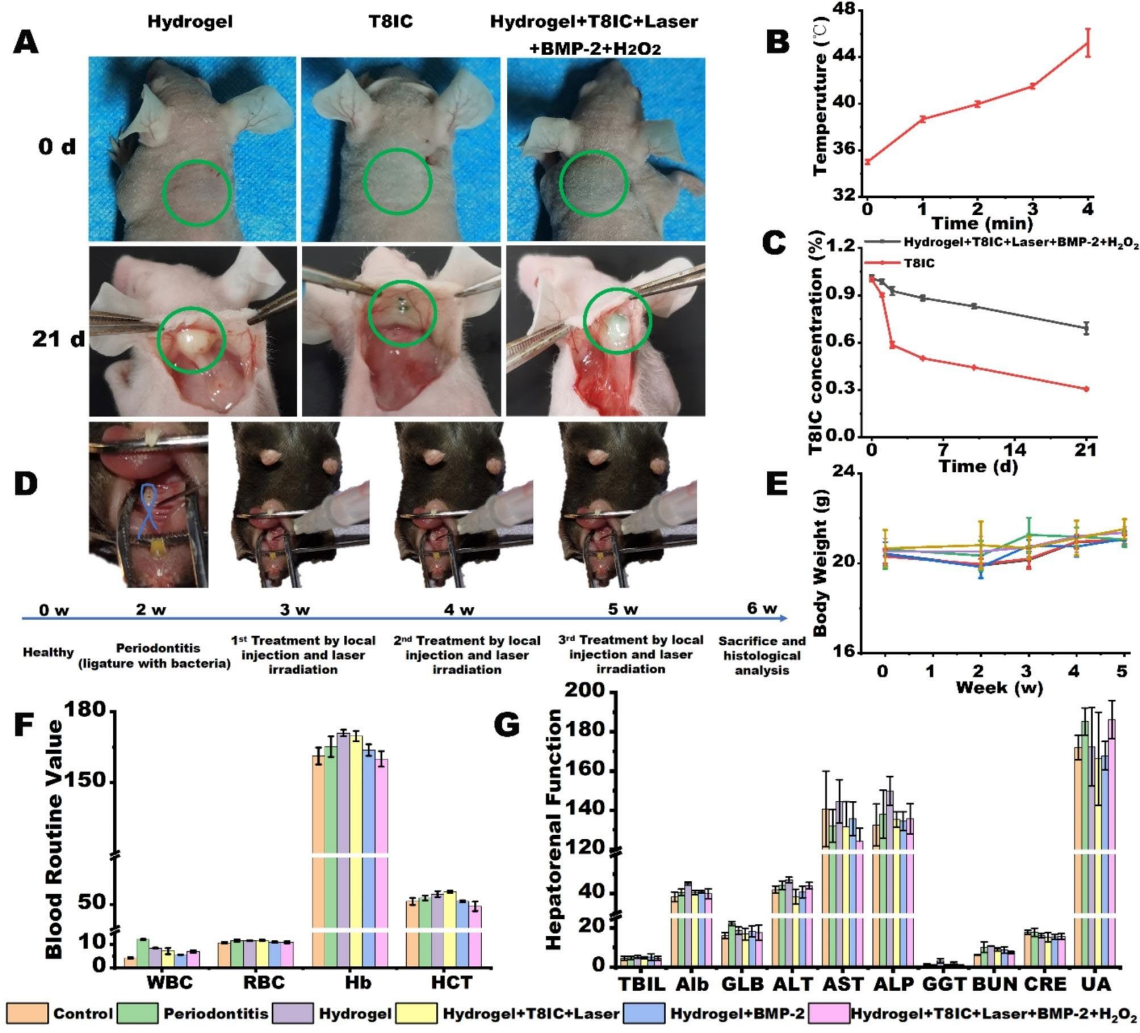
Animal experiments. **A**: 0 d: the pre-gel was injected subcutaneously in nude mice, 21 d: locally formed hydrogel and residual T8IC NPs were marked by a green circle. **B**: The local body temperature of mice after laser irradiation, min: minute. **C**: Concentration changes of T8IC NPs after subcutaneous injection, d: day. **D**: Schematic illustration of periodontitis model construction and treatment, blue: silk ligature with *P. gingivalis*, w: week. **E**: The changes of body weight in each group during treatment, w: week. **F**: The blood routine examination of mice after treatment. **G**: The hepatorenal function of mice after treatment

In order to monitor the metabolism of Hydrogel + T8IC + Laser + BMP-2 + H_2_O_2_ in the body of BALB/C nude mice, an in vivo fluorescence detector with NIR-II fluorescence scanner (**Figure S3)** was designed and assembled. The emission spectrum of T8IC NPs belongs to NIR-II window, so the fluorescence scanner could detect the residual content of T8IC NPs to monitor the metabolism of Hydrogel + T8IC + Laser + BMP-2 + H_2_O_2_in vivo. The sol-gel transition of chitosan/β-glutamine/gelatin hydrogel occurred in 5 min when the temperature reached to 37 ℃ [15]. After the application of NIR-II phototherapy agents (T8IC NPs and H_2_O_2_) and laser irradiation in the present study, the effect of PTT made the sol-gel transition time shorten to 2 min (45 ℃). Thus, when the hydrogel was applied in vivo, it wouldn’t go back to solution if temperature goes down from 45 ℃ to the body temperature and it is able to perform controlled release of the drugs. As shown in Fig. 6A, after 21 days of subcutaneous injection in the back of the mice and light irradiation for once, the hydrogel formed and kept stable. There was no obvious inflammatory response, implying no toxicity of hydrogels in vivo. The local temperature around the subcutaneous injection in the back of the mice raised with the time of laser irradiation (Fig. 6B). The concentration of T8IC NPs in vivo decreased with time in both groups of Hydrogel + T8IC + Laser + BMP-2 + H_2_O_2_ and T8IC NPs (Fig. 6C). When combined with the hydrogel, T8IC NPs in Hydrogel + T8IC + Laser + BMP-2 + H_2_O_2_ could be maintained with a higher concentration and longer time compared to T8IC NPs alone, which is beneficial to the controlled release of drugs. It was proved that the hydrogel presented with good biocompatibility and encapsulation ability.

The periodontitis model was established on female C57BL/6 mice. Micro-CT was applied to assess the establishment of periodontitis around the maxillary second molar. Considering the possible damage to the periodontal tissue generated from PDT and PTT, the treatment strategy was once a week by periodontal local hydrogel injection and laser irradiation. Figure 6D illustrated the establishment of periodontitis and treatment progress. On one hand, treatment once a week and three times in total could leave time for the periodontal tissue to recover, reduce drug dosage and avoid other possible side effects. On the other hand, it could eliminate periodontal pathogens and promote bone formation to the full extent. The body weight of mice was continuously monitored during the 6-week periodontitis model construction and treatment. There were no obvious changes of the weight after local injection of the hydrogel and laser irradiation (Fig. 6E, P > 0.05**)**. The blood routing test and biochemical examination after treatment was conducted to explore the effect on peripheral blood cells index. WBC, RBC, Hb, HCT and all the indexes representing hepatorenal function (including TBIL, Alb, GLB, ALT, AST, ALP, GGT, BUN, CRE and UA) had no significant difference between the experimental and control groups (Fig. 6F and G, P > 0.05), implying a good biosafety of Hydrogel + T8IC + Laser + BMP-2 + H_2_O_2 _in vivo.

### Osteogenic and anti-inflammatory effectsin vivo

**Fig. 7:**
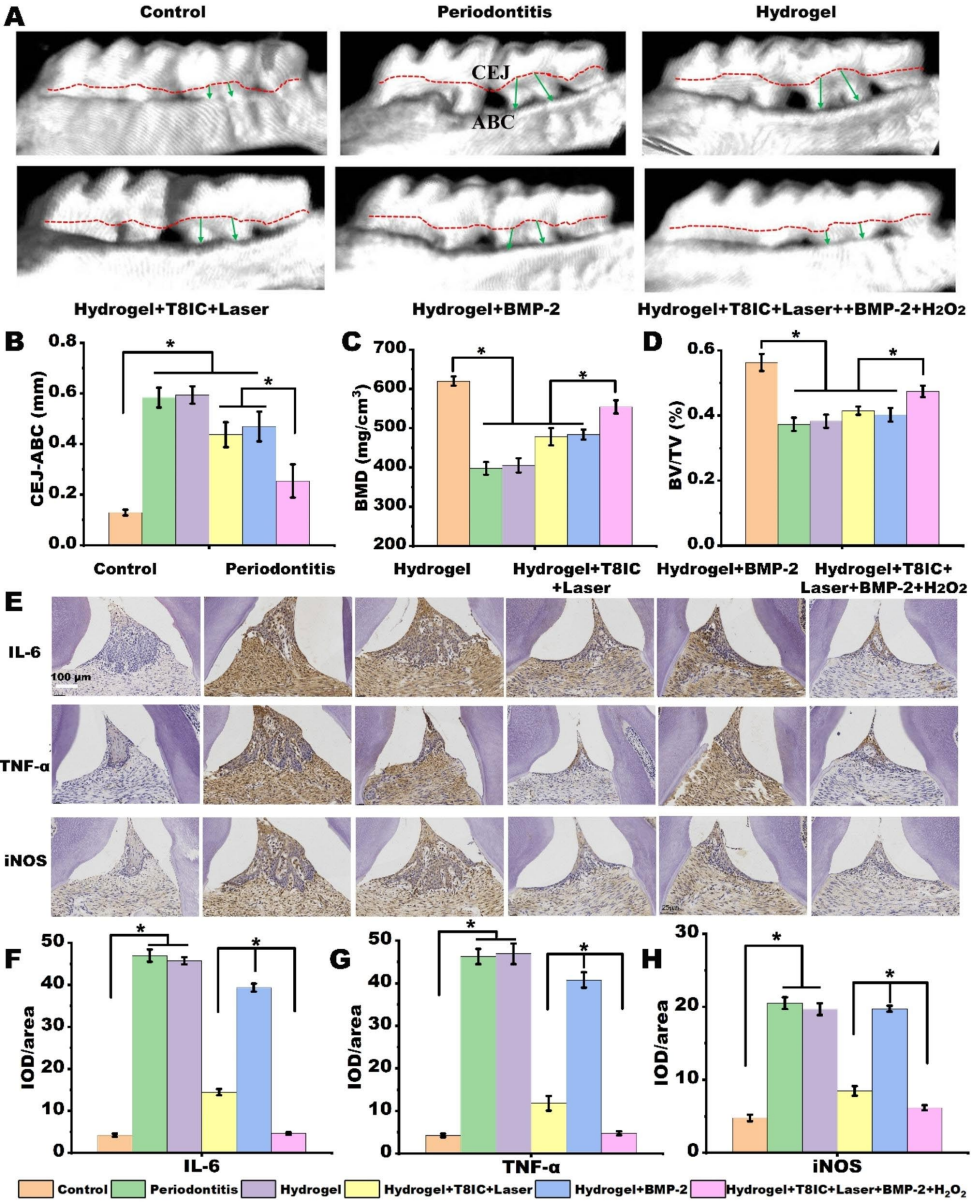
Osteogenic and anti-inflammatory effects *in vivo.***A**: Micro-CT images of maxillary alveolar bone surrounding the maxillary second molars (M2) after the treatment. Green arrow shows distance between cemento-enamel junction and the alveolar bone crest (CEJ-ABC). **B**: Quantitative analysis of CEJ-ABC. **C**: Bone mineral density (BMD, mg/cm^3^). **D**: Bone volume fraction (bone volume/tissue volume, BV/TV, %). **E**: Immunohistochemical staining, scale bar: 50 μm. **F**: IOD/area of IL-6. **G**: IOD/area of TNF-α. **H**: IOD/area of iNOS.

The distance between cemento-enamel junction and the alveolar bone crest (CEJ-ABC) was measured to evaluate the periodontal regeneration [15]. The quantitative analysis of CEJ-ABC showed that there was a significant alveolar bone regeneration in the Hydrogel + T8IC + Laser, Hydrogel + BMP-2 and Hydrogel + T8IC + Laser + BMP-2 + H_2_O_2_.A better bone recovery in the Hydrogel + T8IC + Laser + BMP-2 + H_2_O_2_ was observed when compared with Hydrogel + T8IC + Laser and Hydrogel + BMP-2 (Fig. 7A and B, P < 0.05). Decrease in the bone mineral density (BMD, mg/cm^3^) and bone volume fraction (bone volume/tissue volume, BV/TV, %) was detected after establishment of the periodontitis model in the experimental groups. Both BMD and BV/TV were significantly highest in the Hydrogel + T8IC + Laser + BMP-2 + H_2_O_2_ among the other four experimental groups (Fig. 7C and D, P < 0.05). All those results indicated that hydrogel alone didn’t have the osteogenic effect and hydrogel combined with T8IC + laser or BMP-2 could partially improve the bone regeneration. Significantly, Hydrogel + T8IC + Laser + BMP-2 + H_2_O_2_ exhibited the best therapeutic effect.

Inflammation is a kind of important immune defense. When the tissue is threated by toxins or bacteria, the inflammatory response is initiated and it is a normal state of self-protection and beneficial to human health. However, inflammation is also potentially harmful, if not dealt with in a timely manner, and will cause cytokine storm and other physical dysfunction [66]. The pathogenesis of periodontitis is related to the oral bacteria and the imbalance of immune and inflammatory responses. Under inflammatory conditions, periodontal tissue and immune cells secrete a variety of cytokines (interleukin-1: IL-1, interleukin-6: IL-6, interleukin-10: IL-10, tumor necrosis factor α: TNF-α), which could promote the degradation of connective tissue and accelerate bone destruction [67]. Anti-inflammation is equally important to the bacterial elimination in the treatment of infectious diseases. Mild temperature PTT could inhibit the generation of proinflammatory cytokines (TNF-α, IL-6, IL-1β and IL-10) and the inflammatory stage was converted into the regrowth of new tissue [68, 69].

As shown in Fig. 7A and D, Hydrogel + T8IC + Laser + BMP-2 + H_2_O_2_ exhibited a better osteogenic effect than Hydrogel + BMP-2 and Hydrogel + T8IC + Laser. Furthermore, even without the application of BMP-2, there was bone regeneration in the Hydrogel + T8IC + Laser, implying enhanced PDT and mild PTT may induce bone regeneration through enhancing the anti-inflammation performance, as had been reported [58, 59]. To further confirmation, immunohistochemical staining was performed to analysize the inflammatory state of periodontal tissue. The expression of the proinflammatory cytokines (TNF-α, IL-6 and iNOS) was significantly higher in the group of Periodontitis and Hydrogel (Fig. 7E H**)**. The cytokines in the Hydrogel + T8IC + Laser was lower than that of Hydrogel + BMP-2 and there was a least inflammatory state in the Hydrogel + T8IC + Laser + BMP-2 + H_2_O_2_ (P < 0.05). All those results above implied that PTT and PDT generated by Hydrogel + T8IC + Laser and Hydrogel + T8IC + Laser + BMP-2 + H_2_O_2_ may promote bone regeneration through alleviating the inflammation state.

### Biosafety monitoring

**Fig. 8:**
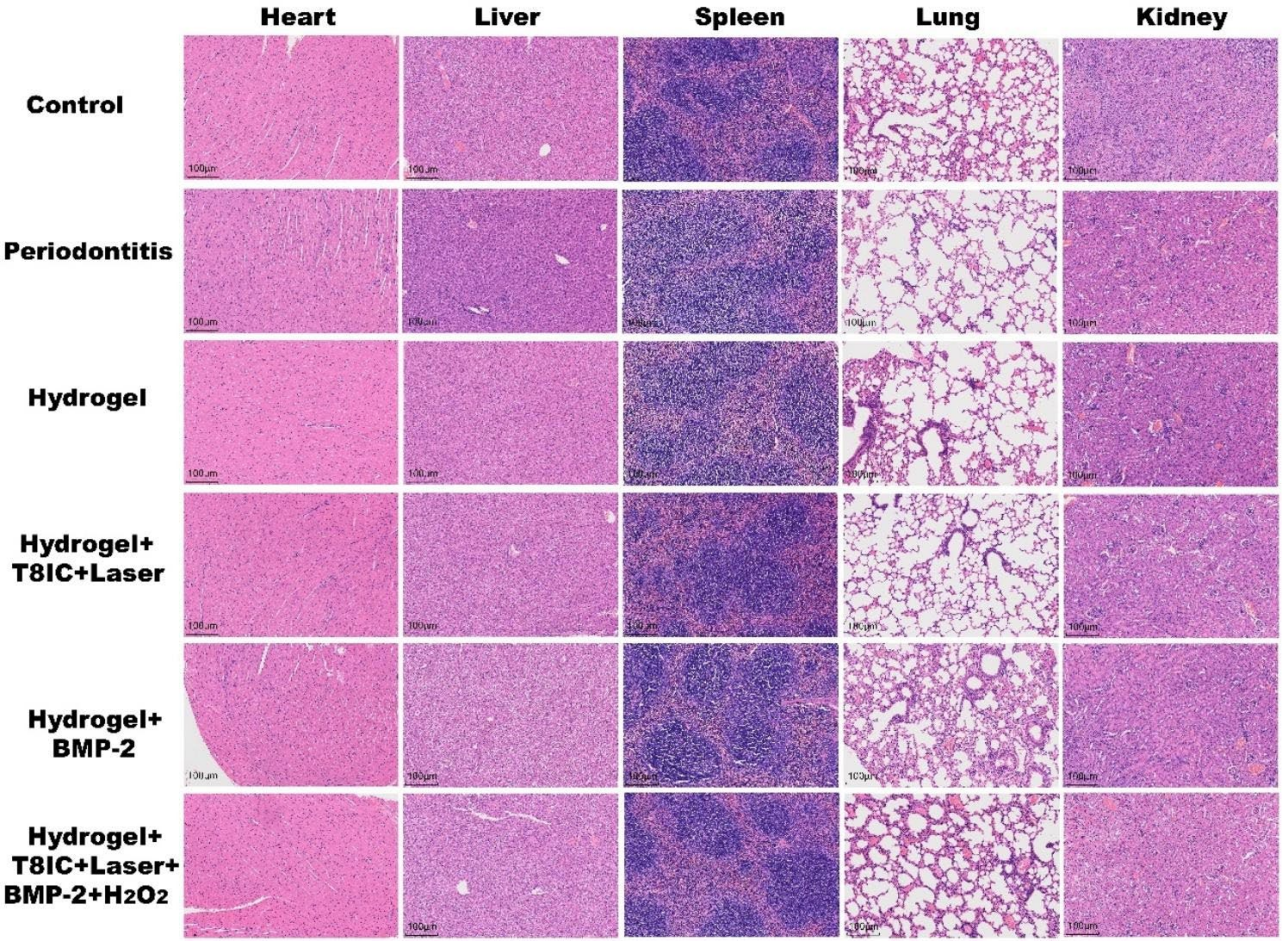
Biocompatibility evaluation after different treatments. H&E staining images of major organs (heart, liver, spleen, lung and kidney), scale bar: 100 μm

The hemolysis assay was performed to detect the biosafety of T8IC NPs in vitro. No hemolysis of red blood cells from mice incubated with different concentrations of T8IC NPs was observed (**Figure S4)**, which validated its biocompatibility. The body weight of mice, blood routing test and biochemical examination were also conducted above to confirmed its biosafety. To further verify their potential toxicity, H&E staining of major organs (including heart, liver, spleen, lung and kidney) was also conducted. The results demonstrated that the cell morphology of major organs was intact and there was no obvious infiltration of inflammatory cells (Fig. 8**).** All results above indicated the excellent biosafety and histocompatibility of Hydrogel + T8IC + Laser + BMP-2 + H_2_O_2_.

## Conclusions

In summary, an injectable and thermosensitive hydrogel with 3D networks was used as drug carrier for controlled release of osteo-inductive agent (BMP-2) and NIR-II phototherapy agents (T8IC NPs and H_2_O_2_). T8IC NPs was prepared by reprecipitation and acted as NIR-II phototherapy agents under 808 nm laser irradiation. To avoid the damage of hyperthermia to surrounding tissues, we promoted PDT through adding H_2_O_2_ to facilitate the antibacterial effect instead of increasing the temperature of PTT. Hydrogel + T8IC + Laser + BMP-2 + H_2_O_2_ incorporated with mild temperature PTT (45 °C), enhanced PDT and sustained release of BMP-2. It was presented with excellent bactericidal effect, osteogenic induction and biosafety both in vitro and in vivo. Even without the application of BMP-2, there was bone regeneration and less expression of the proinflammatory cytokines (TNF-α, IL-6 and iNOS) in the Hydrogel + T8IC + Laser group, implying that PTT and PDT could promote bone regeneration through alleviating inflammation state. In general, this novel approach with synergistic antibacterial effect, anti-inflammation and bone regeneration has a great potential for the treatment of periodontitis in the future.

### Electronic supplementary material

Below is the link to the electronic supplementary material.

Supplementary Material 1
