# Supplementary material for: An injectable and thermosensitive hydrogel with nano-aided NIR-II phototherapeutic and chemical effects for periodontal antibacteria and bone regeneration
Source: J Nanobiotechnology. 2023 Oct 7;21:367. doi: 10.1186/s12951-023-02124-6 (PMC10559606; doi:10.1186/s12951-023-02124-6)
Supplement: Supplementary file 1 — Supplementary Material 1 [file 12951_2023_2124_MOESM1_ESM.docx]

**Supporting Information**


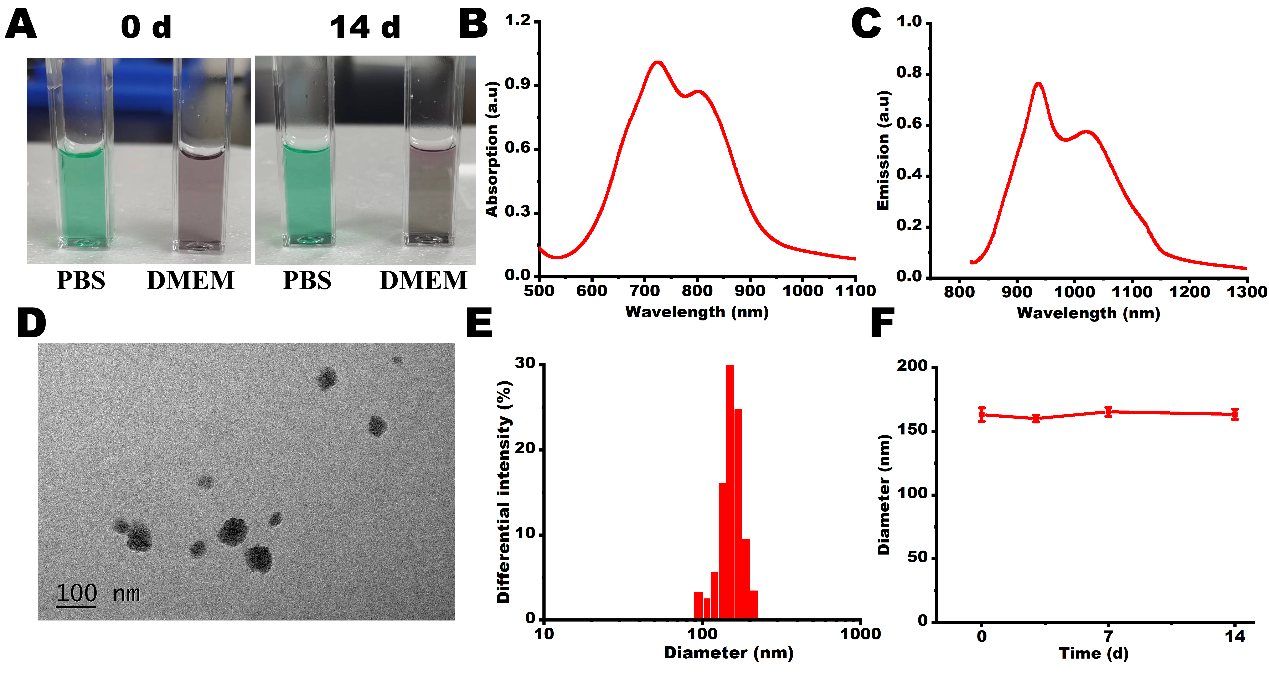


**Figure S1.** Preparation and characterization of T8IC NPs. **A:** Stability of T8IC NPs in PBS and DMEM solutions. **B:** Absorption spectrum of T8IC NPs. **C:** Emission spectrum of T8IC NPs. **D:** Transmission electron microscopy of T8IC NPs. **E:** Particle size of T8IC NPs. **F:** Photothermal stability of T8IC NPs.


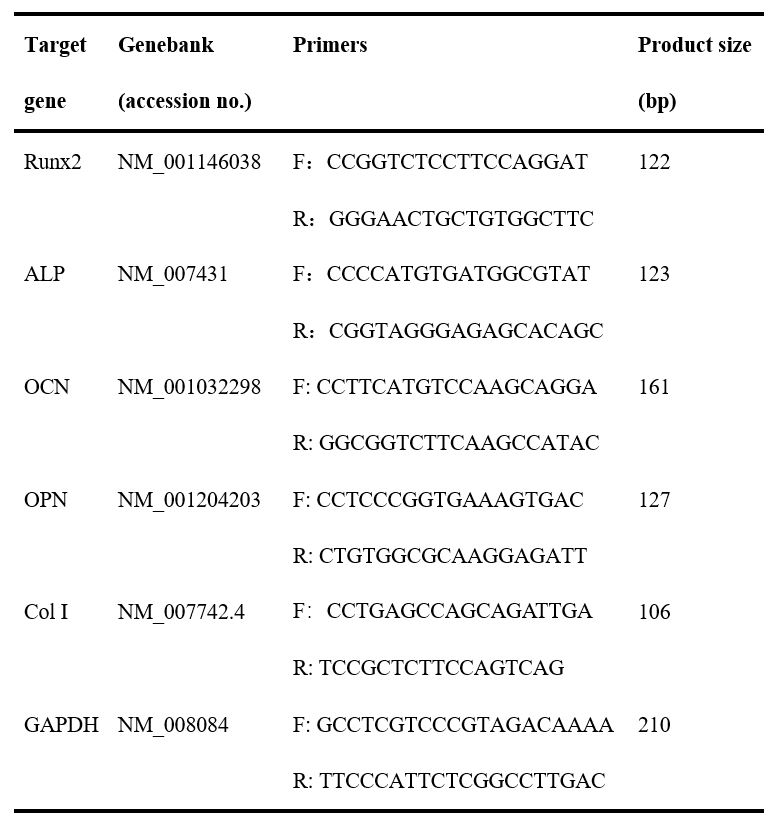


**Figure S2.** Primer sequence.


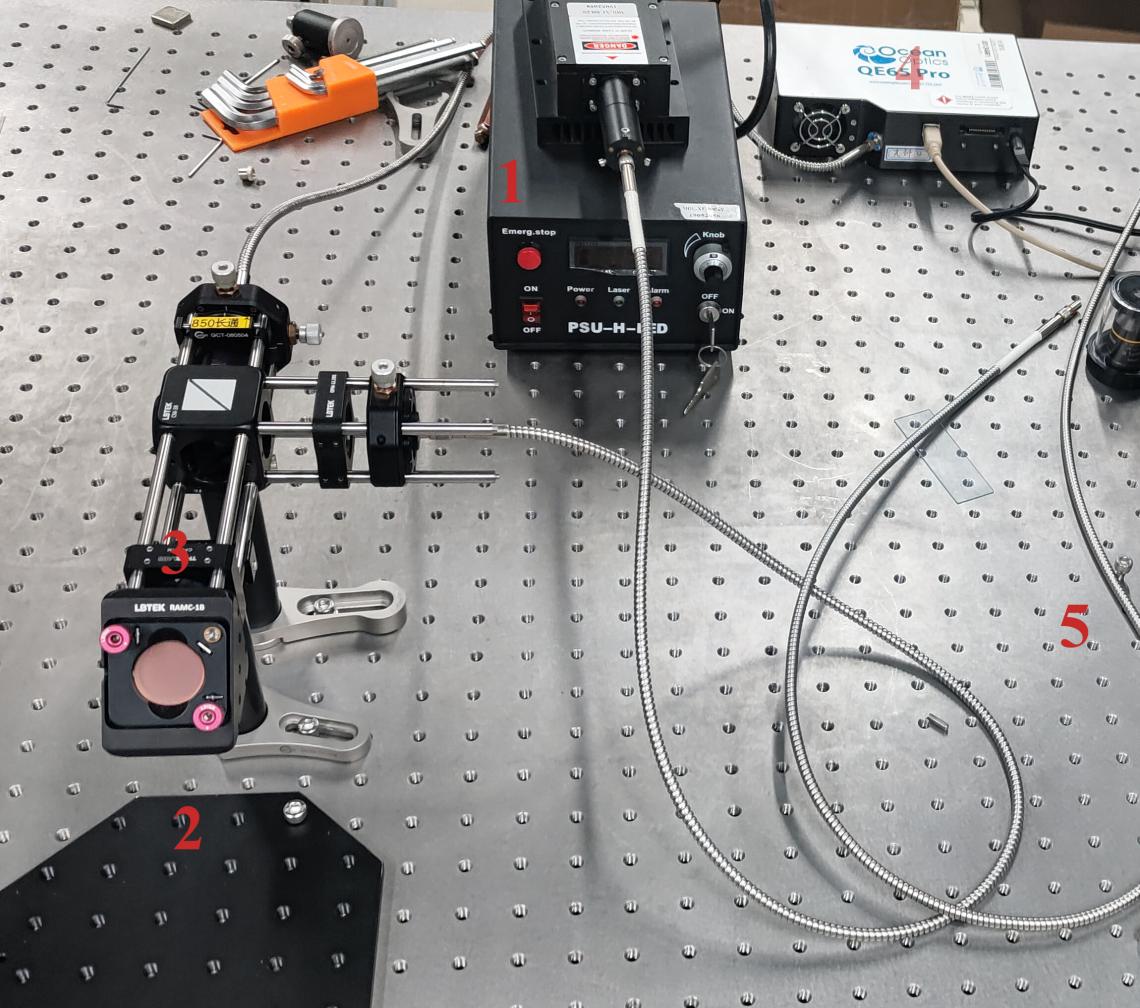


**Figure S3.** Fluorescence detector with near-infrared second-window fluorescence scanner. **1**: Near-infrared second-window laser transmitter. **2**: Objective table, the mice were placed on it after anesthesia. **3-4**: Laser transmitting and receiving converter. **5**: Computer receiving fluorescence signal and converting into digital data.

**
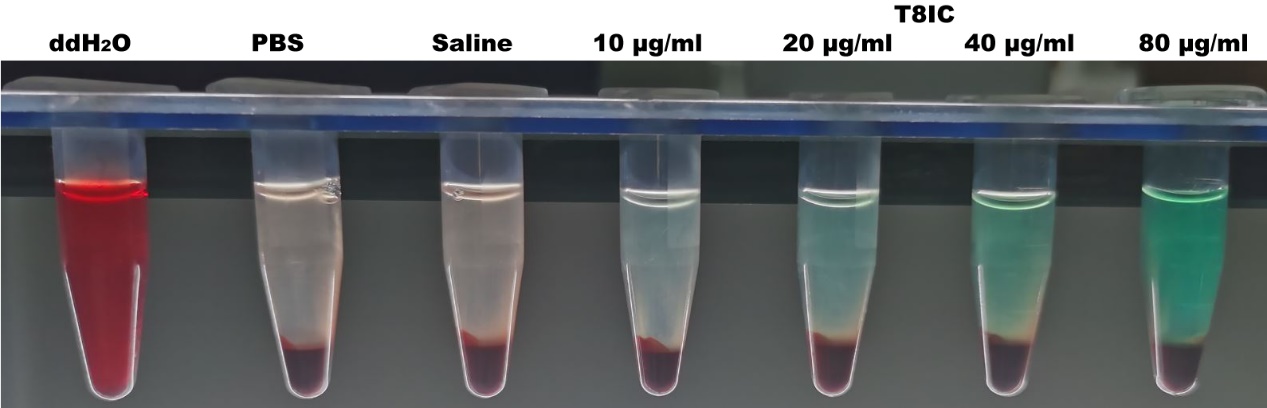
**

**Figure S4.** Hemolytic experiment of different treatments.
